# The Effect of Predictability on Subjective Duration

**DOI:** 10.1371/journal.pone.0001264

**Published:** 2007-11-28

**Authors:** Vani Pariyadath, David Eagleman

**Affiliations:** 1 Department of Neuroscience, Baylor College of Medicine, Houston, Texas, United States of America; 2 Department of Psychiatry, Baylor College of Medicine, Houston, Texas, United States of America; Istituto di Neurofisiologia, Italy

## Abstract

Events can sometimes appear longer or shorter in duration than other events of equal length. For example, in a repeated presentation of auditory or visual stimuli, an unexpected object of equivalent duration appears to last longer. Illusions of duration distortion beg an important question of time representation: when durations dilate or contract, does time in general slow down or speed up during that moment? In other words, what entailments do duration distortions have with respect to other timing judgments? We here show that when a sound or visual flicker is presented in conjunction with an unexpected visual stimulus, neither the pitch of the sound nor the frequency of the flicker is affected by the apparent duration dilation. This demonstrates that subjective time in general is not slowed; instead, duration judgments can be manipulated with no concurrent impact on other temporal judgments. Like spatial vision, time perception appears to be underpinned by a collaboration of separate neural mechanisms that usually work in concert but are separable. We further show that the duration dilation of an unexpected stimulus is not enhanced by increasing its saliency, suggesting that the effect is more closely related to prediction violation than enhanced attention. Finally, duration distortions induced by violations of progressive number sequences implicate the involvement of high-level predictability, suggesting the involvement of areas higher than primary visual cortex. We suggest that duration distortions can be understood in terms of repetition suppression, in which neural responses to repeated stimuli are diminished.

## Introduction

Time is commonly thought to fluctuate in its subjective rate of passage. For example, upon first glance, the second hand of a clock sometimes seems to be frozen in position momentarily before it continues to tick at a normal pace [1], [2]. Perceived duration can be warped by saccades [3], [4], flicker [5], and life-threatening events, which are sometimes anecdotally reported to unfold in slow motion [6], [7]. The neural basis of such distortions remains unknown.

To gain traction on time representation and its plasticity, we turn to a duration distortion that is easily reproduced in the laboratory. Specifically, the first stimulus in a train of repeated presentations is often perceived to have a longer duration than successive stimuli. Participants report duration dilations of as much as 50% in trains of visual stimuli [8], [9], and as much as 15% in trains of auditory stimuli [10]. The above studies proposed that the illusion is a consequence of increased arousal at the first appearance of the stimulus.

Similarly, when an oddball stimulus appears midstream in a repeated presentation of stimuli (auditory or visual), the judged duration of the oddball is overestimated by up to 50% [11], [12]. Tse and his co-authors (2004) proposed an attentional explanation–specifically, that the duration dilation results from an increase in information processed at the time of the oddball due to the deployment of attentional resources. Tse et al (2004) refer to the duration dilation as ‘time's subjective expansion’.

But what does it mean to say that subjective time expands? We here set out to distinguish two hypotheses. In the first, perception works like a movie camera: when one aspect of the scene slows down, everything is slowed down. Thus, if a police car launching off a ramp were filmed using slow-motion photography, it would not only have a longer duration in the air, but also its sirens would blare in a lower pitch, and its lights would blink at a lower temporal frequency. In this case, duration, sound pitch and visual flicker all change hand-in-hand. The second hypothesis, in contrast, supposes that different temporal judgments are generated by different neural mechanisms–and while they often align, they are not required to. Thus, the police car may be judged to have a longer duration in the air, even while the frequencies of its sounds and flickering lights remain unchanged. In this paper, we distinguish these two hypotheses by testing the specific entailments of duration distortions, and in this way are able to directly address the notion of “time's” subjective expansion.

## Materials and Methods

Participants sat 59 cm from a computer monitor and fixated at the center of the screen, and made responses using the keyboard. All participants had normal or corrected-to-normal vision, and were consented according to the procedures of the Institutional Review Board at Baylor College of Medicine.

## Results

### Experiment 1

**Figure 1:**
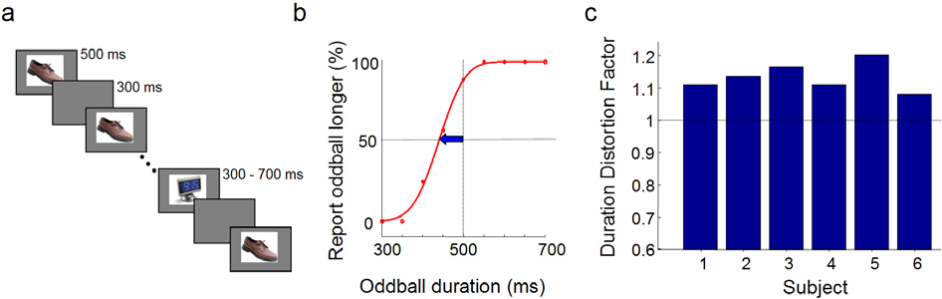
Duration distortion using pictures of everyday objects. (a) Schematic of experiment. Participants reported whether the oddball object embedded at a random position in a stream appeared longer or shorter than the standard (b) Representative data from one participant. The point of subjective equivalence was taken as the 50% point of the psychometric function. (c) The Duration Distortion factor (DDF) is the ratio of the standard duration to the PSE of the oddball for 6 participants.

We began by quantifying the midstream oddball illusion [11]. Six subjects ran 84 trials in which they watched 9 repeated presentations of a photograph with an oddball photograph randomly embedded between the 5th and 8th presentation. Photographs subtended 3.1×3.1° of visual angle and were repeatedly presented at fixation for 500 ms with ISIs of 300 ms. The duration of the oddball varied between 300–700 ms (Figure 1a). After each trial, participants reported whether the oddball was longer or shorter in duration than the ‘standard’ images preceding and succeeding it. The point of subjective equivalence (PSE) for the oddball was taken as the 50% point of the psychometric function (Figure 1b). To measure the dilation, the ‘duration distortion factor' (DDF) was defined as the ratio of the standard duration to the PSE for the oddball (Figure 1c). Note the DDF is defined in the same way as the ‘temporal expansion factor’ from Tse et al (2004).

All 6 participants perceived the oddball to remain on screen longer than the standards. The relative duration distortion averaged 12%. This is consistent with the ∼14.5% previously reported for similar stimuli (flashed geometric shapes [11]).

### Experiment 2

**Figure 2:**
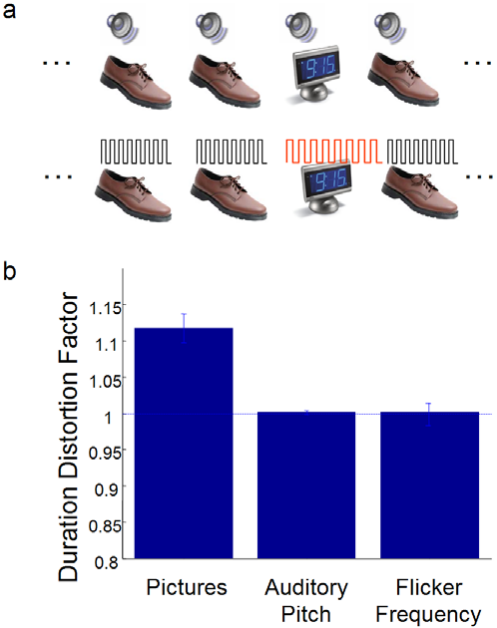
Are accompanying temporal judgments distorted with duration? (a) Experimental details are the same as Figure 1, except that the visual sequence is accompanied by an auditory beep (top), or the photographs are flickered. (b) Mean DDF values for the visual oddball task (averaged from Figure 1c), auditory pitch (n = 6) and flicker rate (n = 6) discrimination tasks.

Having established the basic effect, we next tested whether other temporal measures would be distorted when durations were distorted. For example, if “time subjectively expands” during the visual oddball, does that mean sounds will concomitantly appear to be a lower frequency? Although we take this outcome to be unlikely, it is implicitly embedded in the term “time's expansion”, and has, to our knowledge, never been tested. To this end, our next experimental design was similar to that in Figure 1a, except that now the visual stimuli (including the oddball) were always 500 msec, and each image was accompanied by a 500 msec auditory beep (Figure 2a). The standard photographs were coupled with a beep of 391 Hz, while the beep accompanying the oddball was randomly chosen from one of 9 values between 376–407 Hz. Participants simply reported whether the beep accompanying the oddball was of a higher or lower pitch than the beep accompanying the standards. Participants were not required to make concurrent duration judgments, although they typically volunteered the observation that the oddball seemed to last for a longer duration. Further, it has been shown previously that the accompanying auditory tone is also subject to duration distortions with the appearance of an oddball [13]. To ensure that participants were attending to the visual stimuli and not merely to the auditory stimulus, they were also required to answer an onscreen question about the oddball following each trial: “did you see X in the series?”, where X was replaced by the name of an object. On this identification task, participants performed at an average of 99.14% (data not shown).

Participants showed no difficulty in discriminating the frequency of the beep accompanying the visual oddball from the beep accompanying the standards (Figure 2b, middle bar). We conclude from this result that the oddball illusion is not accompanied by a concurrent distortion of perceived auditory frequency. This indicates that it is not time in general, but only visual durations in particular, that slow during the oddball.

In the next experiment, each visual presentation of the 500 msec standard photographs flickered on and off at 10 Hz, whereas the 500 msec oddball was flickered at a frequency randomly chosen from one of 7 values between 6.25–25 Hz (Figure 2a). Participants reported whether the flicker frequency of the oddball was higher or lower than that of the standards. As before, participants had no trouble accurately discriminating flicker frequencies of the stimuli (Figure 2b, right bar).

Therefore, neither an accompanying auditory stimulus nor visual flicker was distorted in conditions that led to a clear distortion of the perceived duration of the oddball itself (Figure 2b, left bar averaged from data in Figure 1c). While this result might seem obvious from the fact that pitch is hard-coded in the cochlea, and flicker is encoded by low level, dedicated mechanisms, these experiments were necessary to directly address, for the first time, the time-perception-as-movie-camera framework. That framework, commonly implicit in thought and language, necessitates that when perceived durations are dilated, so are other temporal measures–that is, when the police car flies off the ramp, an increased duration is necessarily accompanied by a lowered siren pitch and slower flicker rate. This view of a unified time is implied by terms such as “time's subjective expansion” [11] or “time shrinking” used to describe other duration illusions discussed below [14]–[18]. Our data, however, demonstrate that perceived durations can dilate with no effect on auditory pitch or visual flicker frequency (Figure 2). This allows us to conclude that in the oddball effect, “time” is not subjectively expanded; instead, duration judgments are distorted even while other temporal judgments are not.

### Experiment 3

If subjective time is not slowed down during the appearance of an oddball, what is responsible for distortions of temporal judgments relating to the oddball?

Several authors have suggested that the duration distortion is a consequence of increased attention or arousal triggered by the oddball [8], [11], [12], [19]. For the attentional mechanism, Tse et al (2004) suggest the pacemaker-accumulator model of timing [20], [21] to explain the duration dilation. In this framework, an increase in arousal caused by the appearance of an oddball stimulus leads to a transient increase in the internal clock's tick rate. In consequence, the accumulator collects a larger number of ticks in the same period and duration is perceived as progressing slowly while viewing the oddball.

To understand the role of attention in the duration distortions, we set out to induce a larger duration dilation using more emotionally salient oddballs–i.e., stimuli which activate the amygdala and attract attention more quickly [22], [23]. To this end, we replaced our neutral oddball stimuli with emotionally salient images such as sharks, spiders, snakes, pointed guns, and aggressive dogs. All images were taken from the International Affective Picture System [24]. We used 24 emotionally neutral images that were rated 2.56±0.5 on a scale of 1 to 9 (where 9 represents high arousal), and 24 emotionally salient images that were rated 6.56±0.42.

**Figure 3:**
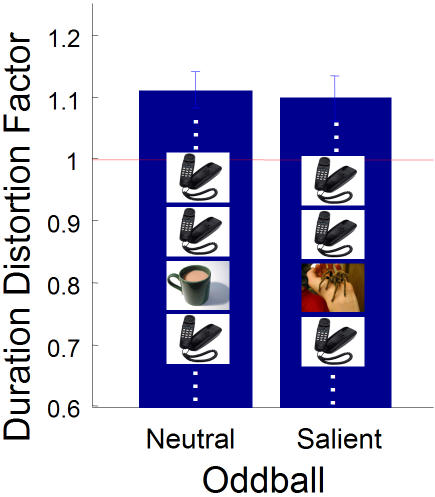
Increasing the emotional salience of the oddball does not increase the effect. DDF values across the two categories of trials–neutral standards with neutral oddball (e.g., coffee cup) and neutral standards with salient oddball (e.g. tarantula). DDF neutral oddball = 1.112, DDF salient oddball = 1.098, p = 0.65. Error bars S.E.M. n = 15.

The oddball effect was unchanged by replacing neutral oddballs with emotionally salient oddballs (Figure 3). This suggests two possibilities: first, since increasing the salience of the oddball failed to increase the duration distortion, it may be that the duration dilation is caused by attentional mechanisms but saturates. Alternatively, it is possible that the oddball effect is not fundamentally an attentional effect.

In considering the latter hypothesis, we began to wonder whether the duration distortion is caused solely by the oddball's unpredictability, irrespective of the subsequent amount of attentional recruitment. To test whether the predictability of the stimulus is responsible for its perceived duration, we turned to the fact that the first stimulus in a repeated series, like an oddball, appears to last longer than the subsequent presentations [8]–[10]. We henceforth refer to this as the *debut effect*.

### Experiment 4

To understand the effect of prediction on duration, we next asked participants to judge the duration of the first stimulus in a visual train and compare it with the stimuli that followed. The experiment involved two interleaved types of trials–in one, the same stimulus was presented four times; in the other, four random stimuli were presented. Participants answered whether the first stimulus was present on screen for a longer or shorter duration than the stimuli that followed. Since the repeated stimuli are more predictable, we hypothesized that the first stimulus would be judged to have lasted longer–perhaps because repeated stimuli would be contracted in duration. In the case of random stimuli, because the succeeding images are not predictable from the first stimulus, no duration distortion would be expected.

**Figure 4:**
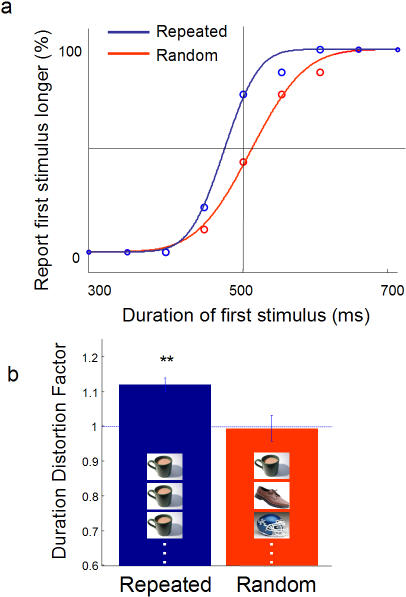
The debut effect disappears with random stimuli. (a) Participants reported whether the first stimulus in a series of repeated or random images appeared longer than the following ones. Representative data from one participant shown. (b) Mean DDF values for the repeated and random series (n = 6). The debut effect only occurs when stimuli are repeated (p<6×10^−4^), implicating the role of predictability. Error bars SEM. n = 8.

With the repeated stimuli, a leftward shift in the psychometric curve was observed (Figure 4), indicating that the first stimulus in the repeated train appeared longer.

However, note that the first stimulus distortion disappears entirely when using random stimuli (Figure 4). Presumably, the use of random stimuli allows each stimulus to act as an unpredicted oddball.

If predictability plays a role in the perceived duration of the stimulus, does this prediction have to be violated at a low level (e.g., involving the edges and the shape of the stimulus) or a high level (e.g., number sequences such as 4…5…6…)? If the latter, this would implicate the involvement of brain areas higher than the primary visual areas. To address this question, we turned to number sequences, since successive numbers differ in shape (analogous to the random images in the previous experiment) but are sequentially predictable.

### Experiment 5

This experiment was similar to experiment 4 but consisted of randomly interleaved trials that either involved (a) a repeated presentation of the number 1 five times, (b) the sequence 1, 2, 3, 4, 5, or (c) a ‘scrambled’ sequence that began with 1 and did not have 2 in its second position (such as 1, 4, 3, 5, 2). Participants answered whether the duration of the first “1” appeared longer or shorter than the stimuli that followed.

**Figure 5:**
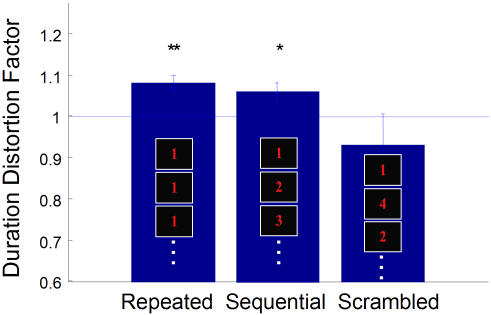
Prediction at higher versus lower levels. Participants made duration discriminations about the first stimulus in a series of repeated, sequential or scrambled number sequences. Both repeated and sequential images had significant duration distortions (p<0.005 and p<0.03, respectively). This distortion was absent in scrambled sequences. Error bars SEM. n = 7.

A similar duration distortion was found in both the repeated and sequential stimuli, but not in the scrambled version (Figure 5). Thus, even though the sequential images were different in terms of edges and shapes, they produced the same effect as a repeated presentation–presumably because the succeeding images could be predicted in sequence. This indicates that the predictability of successive stimuli involves higher cortical areas than the primary visual cortex.

While not statistically significant, note that the scrambled version trended toward a DDF of less than one. While this trend was not predicted by our hypothesis, it may be explained by the recent finding that stimulus *magnitude* affects its perceived duration–for example, a 5 will appear to last longer than a 1 presented for an identical duration [25]. In our scrambled condition, the first number 1 was always followed by a 3, 4 or 5, and thus by comparison may sometimes be judged briefer. In other words, the magnitude effect would tend to push the DDF towards values less than one.

Finally, note that Tse et al (2004) had previously shown that in a series of visually similar stimuli (e.g. figurines of male bodies in different natural poses), a stimulus that belonged to a different category (e.g. figurine of a female body) would be perceived as an oddball and would consequently be dilated in duration. Our findings go beyond that result by demonstrating that even abstract sequences which share little visual similarity can produce such duration distortions.

## Discussion

Our experiments on duration illusions show that subjective time is not a single entity. The oddball and debut illusions involve distortions in duration judgments but do not affect perceived auditory pitch or high visual flicker frequencies. Thus, the oddball and debut illusions do not entail “time's subjective expansion” as was previously hypothesized [11]. Our results suggest that neural systems involved in timing generally work in concert but are nonetheless separable. This is analogous to neural populations which encode motion and position: they tend to work in tandem but can be separated [26], [27]. The motion aftereffect or the waterfall illusion is one such example of perceived motion without a change in position. At the other end of the spectrum lie patients with lesions in MT who are able to perceive change in position but not motion. Analogously, the oddball and debut illusions establish that duration distortions do not entail concurrent distortions of flicker rate or auditory pitch. In other words, time is not one single entity.

We have also shown that the oddball illusion cannot be explained completely by attention, since increasing the emotional salience of the oddball does not bring about a corresponding increase of the duration dilation. This surprising result challenges the conventional viewpoint that duration distortions are caused by deployment of attentional resources when presented with unexpected stimuli [8], [10]–[12]. It still remains a possibility that the attentional effect saturates at ∼14%, although previous experiments using visually expanding stimuli [11] result in duration distortions of 50%, thus weakening the hypothesis of a 14% upper limit. However, an attentional theory alone would not explain our findings using ordinal sequences (Figure 5). Also, duration distortions of the same magnitude occur at timescales too brief to invoke attentional mechanisms (∼80 ms, Pariyadath & Eagleman, submitted).

Our results demonstrate that the duration distortion is caused by the unpredictability of the stimulus, irrespective of the salience (and presumably, the amount of deployed attention) of the oddball. This distortion in duration is found regardless of whether the prediction is made at a low level, i.e. involving edges and shapes or at a higher level involving learned sequences.

**Figure 6:**
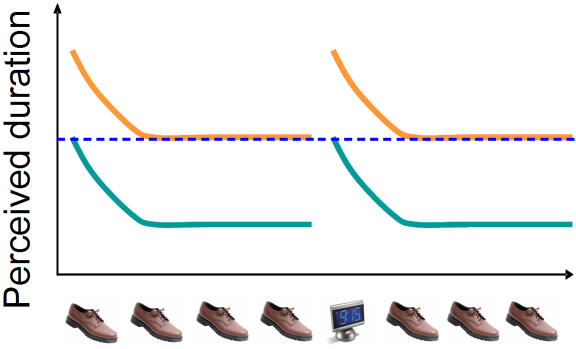
Duration relativity. While the prevailing view of the duration illusion is that the first and oddball stimuli experience duration dilations (red lines), it is equally feasible that the successive, repeated stimuli are experiencing duration contractions (green lines).

Although previous researchers have suggested that the oddball or unexpected stimulus is expanded in duration as compared with the standard (red lines, Figure 6), we suggest here an alternative hypothesis: the oddball's duration may be closer to the physical duration (green lines, Figure 6) while the standards are *contracted* in duration due to their predictability. We base this argument primarily on the neural response to repetition, which we turn to now.

### Repetition suppression and duration

Our findings about duration judgments appear to have a direct parallel with electrophysiologic studies of repetition. In higher cortical areas, neuronal firing rates diminish in response to repeated presentations of stimuli [28]–[30]. Likewise, there is a decrease in amplitude in the ERP signal in response to the second presentation of a stimulus [31]. This stimulus specific adaptation is generally known as *repetition suppression*. fMRI studies have revealed a similar decrease in the BOLD response to repeated presentations of stimuli as compared with the response to novel stimuli [32]. Behaviorally, repetition suppression has been linked to repetition priming or a decrease in reaction time to respond to repeated or familiar stimuli [33], [34].

Suppression of the neural response potentially allows the system to save resources and might reflect a more efficient representation [35]. Suppressing the neural response to familiar stimuli could also allow novel stimuli to more easily obtain attentional resources [36].

We suggest that these changes in the neural response to repeated stimuli map directly onto the perceived duration of the stimuli: the conditions that lead to a suppressed neural response are the same as those that lead to a reduction in perceived duration. If the parallel between neural response and subjective duration is meaningful, this may support the hypothesis that successive stimuli are contracted in duration (lower traces, Figure 6), rather than the oddballs being expanded.

Our results with number sequences (Figure 5) suggest that the neural responses to *predicted* stimuli are suppressed [37] as though they were actually experienced. In other words, the neural response to the successive stimulus in an ordinal sequence would be suppressed, thereby contracting its perceived duration.

### Duration distortion by any other name

Note that the debut illusion has appeared in the literature under various guises. For example, in the “stopped clock illusion”, the second-hand appears to be momentarily frozen when one first looks at a clock's face. This phenomenon, also known as chronostasis, was initially attributed to perceptual ‘filling-in’ during a saccade [1]. However, the effect has also been demonstrated in the auditory [10] and tactile [38] modalities, making saccades an insufficient explanation for the duration distortion. A more general hypothesis is that the duration distortion is caused by voluntary action preceding the appearance of the stimulus [2]. But this hypothesis, as well, does not suffice, because the illusion can be witnessed even in the absence of any voluntary action [8], [39].

Another illusion in the timing literature is referred to as “the subjective shortening of duration”: when two identical stimuli are presented serially, participants perceive the second one to be briefer [9], [40], [41]. Nakajima and his colleagues describe an essentially identical illusion, which they call “time-shrinking”: when a short interval is preceded by an interval that is up to 100 ms shorter, the duration of the second interval is underestimated [14]–[18]. Parsimony suggests that the above illusions are special cases of the debut effect, and perhaps, we hypothesize, the direct perceptual consequence of repetition suppression. It is easy to demonstrate that the “time-shrinking” illusion disappears upon using two random stimuli instead of a repeated stimulus (data not shown).

The next logical step for this line of research will be to examine cell firing rates in the primary and higher visual areas in response to presentations involving oddballs. Other questions we are working on now include: How does the motor system respond to duration illusions? How exactly does the neural firing rate map onto subjective experience of time passage?

In conclusion, subjective time appears not to be a single entity; instead, it is made up of different timing mechanisms, such as flicker perception and duration perception, which generally work in concert but are separable. As suggested by their strong parallel with repetition suppression, duration illusions caused by unexpected stimuli may be due to a contraction in the perceived duration of predicted stimuli, not a dilation of unexpected stimuli.

## References

[1] Yarrow K, Haggard P, Heal R, Brown P, Rothwell JC (2001). Illusory perceptions of space and time preserve cross-saccadic perceptual continuity.. Nature.

[2] Park J, Schlag-Rey M, Schlag (2003). Voluntary action expands perceived duration of its sensory consequence.. Exp Brain Res.

[3] Eagleman DM (2005). Distortions of time during rapid eye movements.. Nat Neurosci.

[4] Morrone MC, Ross J, Burr D (2005). Saccadic eye movements cause compression of time as well as space.. Nat Neurosci.

[5] Kanai R, Paffen CL, Hogendoorn H, Verstraten FA (2006). Time dilation in dynamic visual display.. J Vis.

[6] Stetson C, Fiesta MP, Eagleman DM (2007). Does time really slow down during a frightening event? PLoS One, in press..

[7] Eagleman DM, Tse PU, Buonomano D, Janssen P, Nobre AC (2005). Time and the brain: how subjective time relates to neural time.. J Neurosci.

[8] Rose D, Summers J (1995). Duration illusions in a train of visual stimuli.. Perception.

[9] Kanai R, Watanabe M (2006). Visual onset expands subjective time.. Perception & psychophysics.

[10] Hodinott-Hill I, Thilo KV, Cowey A, Walsh V (2002). Auditory chronostasis: hanging on the telephone.. Curr Biol.

[11] Tse PU, Intriligator J, Rivest J, Cavanagh P (2004). Attention and the subjective expansion of time.. Perception & psychophysics.

[12] Ulrich R, Nitschke J, Rammsayer T (2006). Perceived duration of expected and unexpected stimuli.. Psychological Research.

[13] van Wassenhove V, Buonomano DV, Shimojo S, Shams L (2006). Altering subjective time perception within and across senses. In Society for Neuroscience. (Atlanta, GA)..

[14] Arao H, Suetomi D, Nakajima Y (2000). Does time-shrinking take place in visual temporal patterns?. Perception.

[15] Nakajima Y, ten Hoopen G, Hilkhuysen G, Sasaki T (1992). Time-shrinking: a discontinuity in the perception of auditory temporal patterns.. Perception & psychophysics.

[16] Nakajima Y, ten Hoopen G, Sasaki T, Yamamoto K, Kadota M (2004). Time-shrinking: the process of unilateral temporal assimilation.. Perception.

[17] Sasaki T, Suetomi D, Nakajima Y, ten Hoopen G (2002). Time-shrinking, its propagation, and Gestalt principles.. Perception & psychophysics.

[18] ten Hoopen G, Hartsuiker R, Sasaki T, Nakajima Y, Tanaka M (1995). Auditory isochrony: time shrinking and temporal patterns.. Perception.

[19] Ranganath C, Rainer G (2003). Neural mechanisms for detecting and remembering novel events.. Nature reviews.

[20] Treisman M (1963). Temporal discrimination and the indifference interval: Implications for a model of the ‘internal clock’.. Psychological Monographs.

[21] Gibbon J, Malapani C, Dale CL, Gallistel C (1997). Toward a neurobiology of temporal cognition: advances and challenges.. Current opinion in neurobiology.

[22] Davis M, Whalen PJ (2001). The amygdala: vigilance and emotion.. Molecular Psychiatry.

[23] Holland PC, Gallagher M (1999). Amygdala circuitry in attentional and representational processes.. Trends Cogn Sci.

[24] Lang PJ, Bradley MM, Cuthbert BN (2005). International Affective Picture System (IAPS): Affective ratings of pictures and instruction manual. (Gainesville, Florida: University of Florida)..

[25] Xuan B, Zhang D, He S, Chen X (2007). Larger stimuli are judged to last longer.. Journal of Vision.

[26] Eagleman DM (2007). Fractionating time: the many facets of time perception and their neural underpinnings. Current Directions in Psychology *In press*..

[27] Ross J, Burr D, Morrone C (1996). Suppression of the magnocellular pathway during saccades.. Behavioural brain research.

[28] Fahy FL, Riches IP, Brown MW (1993). Neuronal activity related to visual recognition memory: long-term memory and the encoding of recency and familiarity information in the primate anterior and medial inferior temporal and rhinal cortex.. Exp Brain Res.

[29] Miller EK, Desimone R (1994). Parallel neuronal mechanisms for short-term memory.. Science.

[30] Li L, Miller EK, Desimone R (1993). The representation of stimulus familiarity in anterior inferior temporal cortex.. Journal of neurophysiology.

[31] Grill-Spector K, Henson R, Martin A (2006). Repetition and the brain: neural models of stimulus-specific effects.. Trends in Cognitive Sciences.

[32] Henson RNA, Rugg MD (2003). Neural response suppression, haemodynamic repetition effects, and behavioural priming.. Neuropsychologia.

[33] Dehaene S, Naccache L, Cohen L, Le Bihan D, Mangin J (2001). Cerebral mechanisms of word masking and unconscious repetition priming.. Nat Neurosci.

[34] Orfanidou E, Marslen-Wilson WD, Davis MH (2006). Neural response suppression predicts repetition priming of spoken words and pseudowords.. Journal of cognitive neuroscience.

[35] Wiggs CL, Martin A (1998). Properties and mechanisms of perceptual priming.. Current opinion in neurobiology.

[36] Desimone R, Duncan J (1995). Neural Mechanisms of Selective Visual Attention.. Annual Review of Neuroscience.

[37] Rao RP, Ballard DH (1999). Predictive coding in the visual cortex: a functional interpretation of some extra-classical receptive-field effects.. Nat Neurosci.

[38] Yarrow K, Rothwell JC (2003). Manual chronostasis: tactile perception precedes physical contact.. Curr Biol.

[39] Alexander I, Thilo KV, Cowey A, Walsh V (2005). Chronostasis without voluntary action.. Exp Brain Res.

[40] Wearden JH, Ferrara A (1993). Subjective shortening in humans' memory for stimulus duration.. Q J Exp Psychol B.

[41] Wearden JH, Parry A, Stamp L (2002). Is subjective shortening in human memory unique to time representations?. Q J Exp Psychol B.

